# Simultaneous Combined Myositis, Inflammatory Polyneuropathy, and Overlap Myasthenic Syndrome

**DOI:** 10.1155/2016/6108234

**Published:** 2016-12-01

**Authors:** Stéphane Mathis, Laurent Magy, Philippe Corcia, Karima Ghorab, Laurence Richard, Jonathan Ciron, Mathilde Duchesne, Jean-Michel Vallat

**Affiliations:** ^1^Department of Neurology, Nerve-Muscle Unit, CHU Bordeaux (Groupe Hospitalier Pellegrin), place Amélie-Raba-Léon, 33000 Bordeaux, France; ^2^Department of Neurology, Centre de Référence “Neuropathies Périphériques Rares”, CHU Limoges, 2 avenue Martin Luther King, 87042 Limoges, France; ^3^Department of Neurology, CHU Bretonneau, 2 boulevard Tonnellé, 37044 Tours, France; ^4^Department of Neurology, CHU Poitiers, 2 rue de la Milétrie, 86021 Poitiers, France

## Abstract

Immune-mediated neuromuscular disorders include pathologies of the peripheral nervous system, neuromuscular junction, and muscles. If overlap syndromes (or the association of almost two autoimmune disorders) are recognized, the simultaneous occurrence of several autoimmune neuromuscular disorders is rare. We describe two patients presenting the simultaneous occurrence of inflammatory neuropathy, myositis, and myasthenia gravis (with positive acetylcholine receptor antibodies). For each patient, we carried out a pathological analysis (nerve and muscle) and an electrophysiological study (and follow-up). To our knowledge, this is the first description of such a triple immune-mediated neuromuscular syndrome. We compared our observations with a few other cases of simultaneous diagnosis of two inflammatory neuromuscular disorders.

## 1. Introduction

Neuromuscular disorders are a heterogeneous group of diseases affecting the peripheral nervous system (including anterior horn cells, nerve roots, plexus, and peripheral nerves), the neuromuscular junction, and muscles. These different entities are well described, and the clinical manifestations and some hallmark signs usually help to make the diagnosis [1]. However, such a diagnosis may sometimes be difficult in an atypical presentation or overlap syndrome: for example, some entities can combine myopathy and neuropathy, such as “critical illness neuromuscular dysfunction.” We report here two patients with a subacute presentation of simultaneous myasthenia gravis, inflammatory polyneuropathy, and myositis, and we discuss this rare occurrence of immune-mediated neuromuscular disorders.

## 2. Case Reports

### 2.1. Patient 1

This 67-year-old woman complained of severe asthenia, loss of weight, and overall muscular weakness for five months. Her medical history only showed osteoporosis (without any fracture). On clinical examination we found drop head, bilateral facial weakness, and also swallowing, breathing, and speech troubles (these symptoms were present for two months). We observed a generalized decrease of the muscular strength: the motor weakness was proximal and distal in the four limbs but predominantly in the upper limbs (Medical Research Council (MRC): grade 3 in the upper limbs and grade 4 in the lower limbs). Amyotrophy was moderate and restricted to the hands. She also complained of paresthesia in her hands (for five months), but we found neither sensory deficit nor ataxia. Deep tendon reflexes were weak in the upper limbs but normal in the lower limbs. We observed no cramp or fasciculation. No pyramidal or cerebellar signs were found. She presented a Raynaud phenomenon for one month, but no cutaneous abnormality was observed. She also had a deformity of the hand joints (distal and proximal interphalangeal joints) but without any inflammatory sign and a kyphoscoliosis. We found neither adenopathy nor organomegaly. The cardiovascular and pulmonary examinations were normal.

Ancillary tests showed a high level of creatine kinase (ranging between 400 and 1200 IU/L) and C-reactive protein (91 mg/L) and mild hepatic cytolysis (alanine aminotransferase: 87 U/L; aspartate aminotransferase: 66 U/L). No monoclonal gammopathy was present. The immunological tests showed a positivity of antinuclear factors (titer > 1/640), but anti-double stranded DNA, anti-SM, anti-nucleosome, and anti-cardiolipin antibodies were negative; no cryoglobulinemia was detected. Anti-glycolipid, anti-MAG, and anti-neuronal antibodies were negative, but anti-acetylcholine receptor (anti-AchR) antibodies were positive (14.4 nmol/L; reference range < 0.2 nmol/L). Thyroid stimulating hormone (TSH) level was normal. The various serologies (HIV, hepatitis B and hepatitis C,* Borrelia burgdorferi*, Herpes, VZV, and HTLV) were negative. On lumbar puncture, no cell, no infection, and a normal protein (41 g/dL) and glucose (64 g/dL) levels were found in cerebrospinal fluid (CSF). A whole-body CT-scan did not detect any thymoma or underlying cancer or infection. The electrophysiological study showed decreased CMAP (compound muscle action potentials) amplitude in the upper limbs, with normal motor nerve conduction velocities, distal latencies, and F-wave latencies in the four limbs (Table 1). We also observed moderate decreased SNAP (sensory nerve action potentials) amplitude in the upper limbs and severe reduction in the lower limbs (Table 1). In the upper limbs, needle electromyography showed early recruitment with small and spiky motor units on the deltoid muscles (myogenic pattern) but also a reduced recruitment of the motor units in first dorsal interosseous muscles (neurogenic pattern); there was no spontaneous activity. Repetitive nerve stimulation testing was performed and showed a marked decremental response at 3 Hz stimulation in genioglossus (−48.1%) and also right (−23.7%) and left (−24.3%) abductor digiti minimi. Sural nerve and deltoid muscle biopsies were performed.

This patient has been treated with a cure of intravenous immunoglobulins (IVIg: 0.4 g per day during 5 days) before starting oral steroids (1 mg/kg/day) and then tapering over three months; acetylcholinesterase inhibitors were also added. After 6 months, we observed a dramatic improvement with MRC score graded 5 (proximal and distal) in the four limbs (except on quadriceps: 4/5), with no drop head (but a mild difficulty of the flexion of the head against resistance) and no facial weakness, but still some paresthesia in the feet. Two years after the onset of the symptoms, we observed electrophysiological improvement (amplitude of potentials) on both motor and sensory nerves (Table 1); however, there was still some slowing of motor nerve conduction velocities, demonstrating a mild demyelinating process.

### 2.2. Patient 2

This 79-year-old man complained of generalized weakness for three months. His medical history included shoulder pain and acromioclavicular joint dislocation (seven years earlier); eight years earlier, he presented myocardial infarction (he has been treated by aspirin, statins, and antihypertensive drugs). The first symptoms were lumbar pain and muscular weakness, with progressive worsening for three months although the patient was still autonomous and able to walk. The motor testing found proximal weakness in the four limbs (MRC: grade 3) but no distal weakness; we also observed moderate atrophy of quadriceps and pectoral muscles (without severe muscular pain). Deep tendon reflexes were absent in the lower limbs and weak in the upper limbs. Some difficulties in swallowing were reported (for three months), but he presented no other cranial nerve symptom. He did not complain of any paresthesia, and we did not observe any sensory impairment (in all modalities). There was no pyramidal sign.

The ancillary tests showed a moderate increase of creatine kinase (249 U/L) with normal c-reactive protein (2 mg/L) and also a high level of cholesterol (total cholesterol was 2.36 g/L, and LDL-cholesterol was 1.58 g/L). Antinuclear factors were positive (titer: 1/320), as were anti-DNA antibodies (55 U/L), and anti-SM, anti-nucleosome, and anti-cardiolipin antibodies were negative; no cryoglobulinemia was detected. Anti-glycolipid, anti-MAG, and anti-neuronal antibodies were negative; anti-AchR antibodies were positive (16 nmol/L; reference range < 0.2 nmol/L). There was no monoclonal gammopathy. The spinal MRI was normal (except for moderate lumbar and cervical arthrosis), and we found no thymoma on thoracic CT-scan. The electrophysiological study showed reduced CMAP amplitude in the lower limbs (with increased F-wave latencies but normal nerve conduction velocities) and only a bilateral carpal tunnel syndrome in the upper limbs (Table 1). We also observed reduced SNAP amplitude in the lower limbs (Table 1). In the lower limbs, needle electromyography showed early recruitment with spiky motor units on the quadriceps muscles (myogenic pattern) but also a reduced recruitment of the motor units in tibialis anterior (neurogenic pattern); there was no spontaneous activity. Repetitive nerve stimulation showed a marked decremental response at 3 Hz stimulation in the trapezius muscles (−20%). Sural nerve and deltoid muscle biopsies were performed.

This patient was treated with three monthly doses of IVIg (0.4/g/day for 5 days) and oral steroids (1 mg/kg/day; tapering after 3 months); acetylcholinesterase inhibitors were also added. After 6 months, we observed a moderate improvement in the four limbs with MRC score graded 4 in proximal and still 5 in distal. Four years after the onset of the first symptoms, we observed a mild decrease of amplitude of both CMAP and SNAP (Table 1).

### 2.3. Pathological Findings

After informed consent, nerve and muscle biopsies were performed in the two patients and processed as described elsewhere [35].

**Figure 1:**
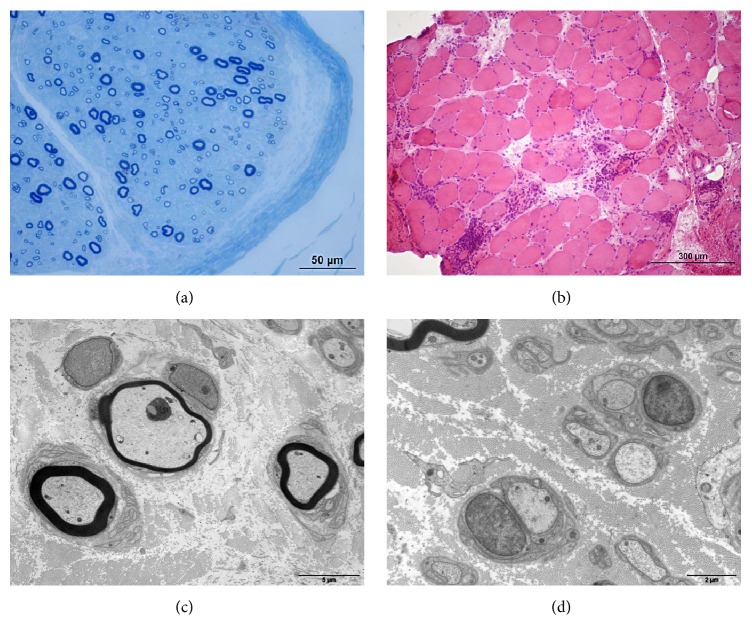
Pathological findings in patient 1. (a) Transverse semithin section of sural nerve stained with Toluidine Blue: there is a moderate loss of myelinated fibers. Only a few fibers have a too thin myelin sheath compared to their axonal diameter. (b) Frozen section of deltoid muscle stained with hematein-eosin showing important infiltrates of mononuclear cells; the immunostaining confirmed that these cells were mostly T cells and macrophages. (c) Electron micrograph of a sural nerve section showing two fibers surrounded by onion bulb formation and a fiber with a thin myelin sheath. (d) Electron micrograph of a sural nerve section showing an axon that has been completely demyelinated.

**Figure 2:**
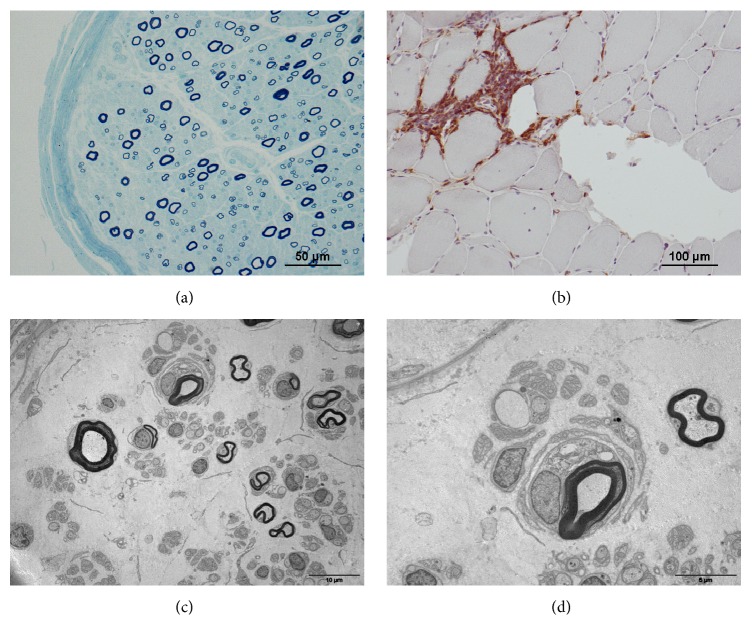
Pathological findings in patient 2. (a) Transverse semithin section of a sural nerve stained with Toluidine Blue: there is a moderate loss of myelinated fibers; several fibers have a too thin sheath compared to their axonal diameter. (b) Frozen section of a deltoid muscle stained with anti-CD45 antibody showing the presence of T cell infiltrates. (c) Electron micrograph of a sural nerve section showing a myelinated fiber which is surrounded by an onion bulb formation. (d) Same field as in (c) but at higher magnification.

On sural nerve of both patients, we observed a pathological pattern of primary demyelinating polyneuropathy, with a moderate loss of myelinated fibers, too thin myelin sheath, and also “onion bulb” formations (Figures 1 and 2). No pattern of vasculitis or amyloid deposit was found and no clonal cell was seen on immunostaining.

Deltoid muscle biopsy of both patients evidenced a classical pattern of inflammatory myopathy. The immunostaining confirmed the presence of inflammatory infiltrates (mainly T cells), without clonal cells (Figures 1 and 2). No amyloid deposits or vasculitis was found in muscles.

### 2.4. Method for the Review of the Literature

We searched MEDLINE, Scopus, and Google Scholar for case reports and case series of patients with at least two simultaneous dysimmune neuromuscular disorders (inflammatory polyneuropathy, myasthenia gravis, or myositis) published since 1975. We include all cases with sufficient clinical, electrophysiological, biological, or pathological data to perform a diagnosis of one of these three dysimmune neuromuscular disorders.

## 3. Discussion

Idiopathic inflammatory muscle diseases (or myositis) are rare (annual incidence range from 2.1 to 7.7 cases per million) and represent a heterogeneous group of acquired myopathies comprising pure polymyositis, pure dermatomyositis, necrotizing myopathy, inclusion body myositis, overlap myositis, and myositis-specific autoantibodies (and associated diseases), according to the usual classification [36]. They are characterized by mainly proximal motor weakness (acute, subacute, or chronic) of the four limbs on clinical examination and inflammatory signs on muscle biopsy (with the presence of T cells, macrophages, dendritic cells, B cells, and plasma cells in the muscle tissue) [37]. More than half of the myositis is associated with the presence of autoantibodies [36]. These pathological and biological features suggest that immune mechanisms are involved in their pathogenesis [36]. Myositis patients can also develop additional autoimmune diseases, as seen in the “overlap syndromes” corresponding to the association of at least two different connective tissue diseases (such as the association of systemic sclerosis and myositis, also named “scleromyositis”) [38]. Moreover, patients with idiopathic inflammatory myopathies may present various extramuscular signs such as skin manifestation (in dermatomyositis), cardiac disturbances, gastrointestinal disorders, pulmonary symptoms, or general symptoms (such as fever or Raynaud phenomenon) [39]. However, among all these manifestations, concomitant neurological and neuromuscular disorders have been rarely reported.

The association of myositis and neuritis was first described by Senator at the end of the nineteenth century [40]. The term of “neuromyositis” was given to this entity including acute or subacute occurrence of neuropathic signs (distal weakness and sensory disturbances, paresthesia, absent Achilles reflexes, and sometimes ataxia) and muscular signs (muscle hypertrophy then amyotrophy and myalgia), with pathological sign of inflammation in nerves and muscles [41]. Since that time, a decade of cases of “neuromyositis” were reported, usually with inflammatory signs in nerves (perineural infiltrates of lymphocytes or neutrophils) [42–44]; however, some patients may present nonspecific axonal neuropathy with no inflammation in the nerve [10], and recent reports also described nerve vasculitis [7, 18, 25]. These recent observations suggest that “neuromyositis” could be due to a vasculitic process as evidenced by the overexpression of VEGF (vascular endothelial growth factor) in both nerve and muscle of two patients (in comparison to controls) [18]. We did not observe any sign of vasculitis in nerves and muscles of our patients, but we noted pathological signs of demyelinating neuropathy suggesting that the mechanism of “neuromyositis” could be immune-mediated. However, this association may well represent an incidental combination of myositis and neuritis.

The simultaneous occurrence of Guillain-Barré syndrome (GBS) (as well as Miller-Fisher syndrome) and myasthenia gravis (MG) has also been reported in a few cases [3, 6, 9, 11]. The molecular mimicry between the infectious agents and self-antigens may initiate GBS and MG concurrently, as suggested by Krampfl et al. who showed that antibodies from patients with GBS can cross-react against acetylcholine receptors from mice [45]. It is also known that various infectious agents (such as viruses) may cause myalgias, rhabdomyolysis, and myositis (muscle biopsy showing degeneration and necrosis with overall little inflammatory infiltrates) [46]. In our cases, despite no evidence of infection or glycolipid antibodies, infectious or postinfectious origin could not be ruled out. Moreover, patient 2 has probably more developed acute onset CIDP (a-CIDP) than GBS.

In a recent study, six patients with concurrent MG and inflammatory myopathy (dermatomyositis or polymyositis) were reported, with also twenty other cases found in the medical literature since 1976: most of these patients had no thymic hyperplasia, and the main myasthenic manifestations were bulbar weakness (83%), limb weakness (83%), ptosis (33%), and diplopia (17%) [5]. The presence of pathological lesions in MG is not recent and was observed early in the beginning of the twentieth century. Carl Weigert first described the relationship between hypertrophy of the thymus and MG but also observed lymphocytic infiltrations (“lymphorrhages”) in muscles of patients with MG (that he considered as “metastases of the thymoma”) [47]. Lymphorrhages are found more frequently in patients with thymoma [48]. Other features have also been described in muscles of patients with MG, such as neurogenic muscular atrophy and focal myositis: focal myositis seems to occur chiefly in patients with thymoma [49], whereas neurogenic abnormalities are not linked to thymoma [50]. A relation was also established between lymphorrhages and anti-muscle antibodies [48]. Despite this strong relation between lymphorrhages and thymoma, our two patients presented no thymoma. Finally, myositis and myocarditis may be sometimes considered as an uncommon complication of MG. Some authors have described the occurrence of “nucleated giant cells” (probably due to muscle regeneration following inflammatory degeneration and necrosis) in the skeletal muscles and myocardium of patients with myasthenia and thymoma [51–63], but it was not the case in our patients. One explanation could be the presence of “striational antibodies” (recognizing epitopes on skeletal muscle proteins) detected in the serum of some patients with MG (particularly those against titin, ryanodine receptor, and Kv1.4) [64]. However, because such antibodies are not routinely tested, we were unable to test their presence in our patients.

The simultaneous occurrence of three autoimmune neuromuscular disorders is rare, and we found only one similar observation in the literature (a 71-year-old man who presented polymyositis, MG, and neuropathy in a context of T cell lymphoma) [20] (Table 2): electrophysiological features of axonal sensorimotor neuropathy were found, but no nerve biopsy was performed; MG was confirmed by a positivity of AchR antibodies (along with a thymic mass); muscle biopsy was consistent with a chronic inflammatory myopathy (no T cell infiltrates were observed). This observation is similar to ours (but without certainty of inflammation of the nerves), with an immune-mediated mechanism. However, the authors have not given any details about the follow-up of the patient. In our patients, we observed a significant improvement after immunosuppressive (steroids), immunomodulatory (IgIV), and acetylcholinesterase inhibitors. The simultaneous diagnosis of two acute/subacute (<3 months) autoimmune neuromuscular disorders is rare but more frequent than the triple association we have observed. Excluding the association of MG-thymoma-myositis-cardiomyositis (because of nonsimultaneous diagnosis, but successive diagnosis), we have found 41 cases in the medical literature: among these cases, the most frequent association was myositis-myasthenia (27 cases), neuropathy-myasthenia (8 cases), and neuropathy-myositis (6 cases) (Table 2). If it was not the case for our patients, thymoma has been found in some cases. Moreover, even if initial screening is negative, the long-term follow-up for the tumor is needed in such cases.

Finally, our cases illustrate the difficulty in diagnosis of neuromuscular disorders in the presence of overlaps. Although a chance association is possible, we suspect that the coexistence of these three inflammatory disorders relies on common immunological mechanisms: it should be borne in mind that a neuromuscular disorder can hide one or two others. Such associations are evidence for an immune-mediated origin of these diseases, but further studies are needed to confirm this hypothesis. These rare occurrences need to be recognized in order to manage these patients appropriately.

## Figures and Tables

**Table 1:** Motor and sensory nerve conduction studies: follow-up of our two patients.

	Nerve	DA	DL	NCV	F	CB
*Patient 1*						
ENMG1 (year 0)						
Motor nerves						
Right	Median	1.46 mV	4.74 ms	56.3 m/s	30 ms	No
	Ulnar	4 mV	4.34 ms	65.8 m/s	31.4 ms	No
	Peroneal	5.2 mV	4.1 ms	46.9 m/s	51.9 ms	No
	Tibial	15 mV	4.58 ms	ND	52.7 ms	ND
Left	Median	2.6 mV	3.4 ms	55 m/s	31.8 ms	No
	Ulnar	3.9 mV	3.87 ms	60 m/s	31 ms	No
	Peroneal	5.2 mV	4.03 ms	41.3 m/s	50.4 ms	No
	Tibial	9.9 mV	4.58 ms	ND	54.8 ms	ND
Sensory nerves						
Right	Median	8.5 *µ*V	—	45.5 m/s	—	—
	Ulnar	5.8 *µ*V	—	57.1 m/s	—	—
	Peroneal	0 *µ*V	—	—	—	—
	Sural	0 *µ*V	—	—	—	—
Left	Median	6.5 *µ*V	—	42.7 m/s	—	—
	Ulnar	8 *µ*V	—	51.6 m/s	—	—
	Peroneal	0 *µ*V	—	—	—	—
	Sural	4 *µ*V	—	36.8 m/s	—	—
ENMG2 (year 1)						
Motor nerves						
Right	Median	2.9 mV	3.55 ms	46.3 m/s	ND	No
	Ulnar	7.5 mV	2.13 ms	57.5 m/s	30.5 ms	No
	Peroneal	7.4 mV	13.2 ms	43.3 m/s	54.1 ms	No
	Tibial	19.4 mV	3.32 ms	ND	51.4 ms	ND
Left	Median	5.8 mV	2.76 ms	55.1 m/s	27 ms	No
	Ulnar	6.5 mV	2.92 ms	71.8 m/s	27.3 ms	No
	Peroneal	4 mV	10.7 ms	42.5 m/s	50.7 ms	No
	Tibial	8.4 mV	3.32 ms	ND	56.1 ms	ND
Sensory nerves						
Right	Median	9 *µ*V	—	49.8 m/s	—	—
	Ulnar	4.9 *µ*V	—	46 m/s	—	—
	Peroneal	0 *µ*V	—	—	—	—
	Sural	2.8 *µ*V	—	44.2 m/s	—	—
Left	Median	9.2 *µ*V	—	33.7 m/s	—	—
	Ulnar	10.6 *µ*V	—	47.9 m/s	—	—
	Peroneal	0 *µ*V	—	—	—	—
	Sural	0 *µ*V	—	—	—	—
ENMG3 (year 2)						
Motor nerves						
Right	Median	4.1 mV	3.24 ms	41.4 m/s	27.2 ms	No
	Ulnar	ND	ND	ND	ND	No
	Peroneal	6.3 mV	3.16 ms	42.6 m/s	48.3 ms	No
	Tibial	15 mV	3.57 ms	35.6 m/s	51.4 ms	ND
Left	Median	8.2 mV	2.92 ms	53.5 m/s	29.4 ms	No
	Ulnar	ND	ND	ND	ND	No
	Peroneal	2.6 mV	3.79 ms	42.8 m/s	40.6 ms	No
	Tibial	10.8 mV	3.9 ms	33.6 m/s	56.3 ms	ND
Sensory nerves						
Right	Median	6.3 *µ*V	—	48.8 m/s	—	—
	Ulnar	ND	—	ND	—	—
	Peroneal	0 *µ*V	—	—	—	—
	Sural	4 *µ*V	—	54 m/s	—	—
Left	Median	7.9 *µ*V	—	47.2 m/s	—	—
	Ulnar	ND	—	ND	—	—
	Peroneal	0 *µ*V	—	—	—	—
	Sural	0 *µ*V	—	—	—	—
*Patient 2*						
ENMG1 (year 0)						
Motor nerves						
Right	Median	7.5 mV	4.2 ms	46.9 m/s	33.2 ms	No
	Ulnar	6.4 mV	2.5 ms	60 m/s	32.4 ms	No
	Peroneal	2.3 mV	5.2 ms	42.3 m/s	60.3 ms	No
	Tibial	2 mV	5 ms	42.4 m/s	63.3 ms	ND
Left	Median	8.2 mV	4.4 ms	45 m/s	32.5 ms	No
	Ulnar	7.8 mV	3.1 ms	62.2 m/s	33.1 ms	No
	Peroneal	2.3 mV	5.2 ms	42.7 m/s	60.8 ms	No
	Tibial	1.3 mV	4.5 ms	44.9 m/s	63 ms	ND
Sensory nerves						
Right	Median	6.5 *µ*V	—	46.3 m/s	—	—
	Ulnar	3.5 *µ*V	—	32 m/s	—	—
	Peroneal	ND	—	ND	—	—
	Sural	4.6 *µ*V	—	40 m/s	—	—
Left	Median	3 *µ*V	—	43.1 m/s	—	—
	Ulnar	3.3 *µ*V	—	40 m/s	—	—
	Peroneal	ND	—	ND	—	—
	Sural	ND	—	ND	—	—
ENMG2 (year 4)						
Motor nerves						
Right	Median	6.3 mV	6.14 ms	45.2 m/s	38.5 ms	No
	Ulnar	7.1 mV	2.65 ms	59.5 m/s	35.5 ms	No
	Peroneal	ND	ND	ND	ND	ND
	Tibial	1.18 mV	5.02 ms	ND	61.6 ms	ND
Left	Median	ND	ND	ND	ND	No
	Ulnar	ND	ND	ND	ND	No
	Peroneal	ND	ND	ND	ND	ND
	Tibial	ND	ND	ND	ND	ND
Sensory nerves						
Right	Median	0 *µ*V	—	—	—	—
	Ulnar	4.1 *µ*V	—	42 m/s	—	—
	Peroneal	ND	—	ND	—	—
	Sural	2.1 *µ*V	—	38.9 m/s	—	—
Left	Median	ND	—	ND	—	—
	Ulnar	ND	—	ND	—	—
	Peroneal	ND	—	ND	—	—
	Sural	ND	—	ND	—	—

CB: conduction block; DA: distal amplitude; DL: distal latency; F: F wave; *µ*V: microVolt; ms: millisecond; m/s: meter/second;  mV: millivolt; NCV: nerve conduction velocity.

**Table 2:** Main characteristics of the patients with concurrent acute/subacute autoimmune neuromuscular disorders diagnosed at the same time (ranked in descending chronological order).

References	Type of NMD			Age	Sex	Description of NMD
	N	M	MG			
Our observations [2]						
Patient 1	•	•	•	67	F	Simultaneous diagnosis of MG (no thymus abnormality), subacute inflammatory neuropathy, and myositis
Patient 2	•	•	•	79	M	Simultaneous diagnosis of MG (no thymus abnormality), subacute inflammatory neuropathy, and myositis
Tanaka & Satomi, 2016 [3]	•		•	69	F	Simultaneous diagnosis of MFS (positivity of anti-GQ1b antibodies) and MG
Seton et al., 2013 [4]		•	•	46	F	Simultaneous diagnosis of PM and MG (with thymoma)
Paik et al., 2014 [5]						
Patient 1		•	•	75	F	Simultaneous diagnosis of DM and MG
Patient 2		•	•	44	M	Simultaneous diagnosis of PM and MG (with thymic mass and lymphoid follicular hyperplasia)
Patient 3		•	•	54	M	Simultaneous diagnosis of PM and MG
Patient 4		•	•	38	F	Simultaneous diagnosis of DM and MG (with thymic mass and lymphoid follicular hyperplasia)
Patient 5		•	•	61	M	Simultaneous diagnosis of PM and MG
Patient 6		•	•	24	F	Simultaneous diagnosis of PM and MG
Belin et al., 2013 [6]	•		•	58	M	Simultaneous diagnosis of GBS and MG
Reimann et al., 2011 [7]	•	•		57	M	Simultaneous diagnosis of myositis and neuropathy with pipestem capillaries and vascular activated complement deposition
Hill et al., 2011 [8]		•	•	67	F	Simultaneous diagnosis of DM and MG
Kung et al., 2009 [9]	•		•	36	F	Simultaneous diagnosis of GBS and MG
Nomura et al., 2010 [10]	•	•		52	F	Simultaneous diagnosis of DM and severe axonal neuropathy (nerve biopsy: axonal atrophy, ovoids, no demyelinating feature, no vasculitis)
Kizilay et al., 2008 [11]	•		•	52	M	Simultaneous diagnosis of GBS and MG
Kraus et al., 2007 [12]	•		•	65	M	Development of MG 10 weeks after GBS
Yoshidome et al., 2007 [13]		•	•	62	F	Simultaneous diagnosis of PM and MG
Avni et al., 2006 [14]		•	•	66	M	MG (with thymoma) diagnosed 2 weeks after treatment for PM (with high level of blood eosinophils)
Shichijo et al., 2005 [15]		•	•	57	M	Simultaneous diagnosis of DM and MG
Farah et al., 2005 [16]	•		•	71	F	Diagnosis of AMSAN two years after a diagnosis of MG
Diaco et al., 2004 [17]		•	•	47	F	Myositis (antisynthetase syndrome) developed in a context of MG
Matsui et al., 2003 [18]						
Case 1	•	•		68	F	Simultaneous diagnosis of DM and inflammatory neuropathy
Case 2	•	•		48	F	Simultaneous diagnosis of DM and inflammatory neuropathy
Van de Warrenburg et al., 2002 [19]		•	•	28	F	Simultaneous diagnosis of DM and MG
Otton et al., 2000 [20]	•	•	•	71	M	Simultaneous diagnosis of MG (with thymoma) sensorimotor neuropathy (no nerve biopsy), and PM
Kornizky et al., 2000 [21]		•	•	69	M	PM in a context of MG
Raschilas et al., 1999 [22]		•	•	66	F	Simultaneous diagnosis of PM and MG (with malignant thymoma)
Kobayashi et al., 1997 [23]		•	•	14	F	PM developed 29 years after MG (in a context of Grave's syndrome)
Ko et al., 1995 [24]		•	•	25	F	Simultaneous diagnosis of PM and MG (with thymoma), with also autoimmune active chronic hepatitis
Vogelgesang et al., 1995 [25]	•	•		9	M	Simultaneous DM and sensorimotor neuropathy (no biopsy)
	•	•		7	F	Simultaneous DM and sensorimotor neuropathy (nerve biopsy: vasculitis)
Hausmanowa-Petrusewicz et al., 1995 [26]		•	•	58	F	DM developed 9 years after MG
Hassel et al., 1992 [27]		•	•	37	M	Simultaneous diagnosis of PM and MG two days after thymectomy (for thymoma)
Carlander et al., 1991 [28]	•		•	45	M	MG with recurrent episodes of GBS
García-Merino et al., 1991 [29]	•		•	68	M	Inflammatory neuropathy (and continuous muscle fiber activity) developed 12 years after a diagnosis of MG (with thymoma)
Behan et al., 1982 [30]		•	•	56	M	Simultaneous diagnosis of PM and MG, with also Hashimoto's thyroiditis, pemphigoid, carcinoma of the bladder, and Norwegian scabies
Boudouresques et al., 1981 [31]	•		•			MG developed 7 months prior to GBS
Davis & Gallai, 1979 [32]		•	•	71	F	MG developed after PM
Vasilescu et al., 1978 [33]						
Case 1		•	•	24	F	Simultaneous diagnosis of DM and MG
Case 2		•	•	46	M	Simultaneous diagnosis of DM and MG
Case 3		•	•	18	F	Simultaneous diagnosis of DM and MG
Case 4		•	•	42	F	Simultaneous diagnosis of DM and MG
De Reuck et al., 1976 [34]		•	•	23	M	Simultaneous diagnosis of PM and MG

AMSAN: acute motor sensory axonal neuropathy; DM: = dermatomyositis; GBS: Guillain-Barré syndrome; M: myositis; MFS: Miller Fisher syndrome; MG: myasthenia gravis; N: acute/subacute neuropathy; NMD: neuromuscular disorder; PM: polymyositis.

## Abbreviations

AchR: Acetylcholine receptor
CMAP: Compound muscle action potential
CSF: Cerebrospinal fluid
CT-scan: Computerized tomography scanner
DNA: Deoxyribonucleic acid
GBS: Guillain-Barré syndrome
IU: International unit
IVIg: Intravenous immunoglobulins
LDL: Light density lipoprotein
MAG: Myelin-associated glycoprotein
MRC: Medical Research Council
MG: Myasthenia gravis
MRI: Magnetic resonance imaging
SNAP: Sensory nerve action potential
VEGF: Vascular endothelial growth factor.
